# Exploring the association between vitamin D levels and dyslipidemia risk: insights from machine learning and generalized additive models

**DOI:** 10.3389/fnut.2025.1618610

**Published:** 2025-08-11

**Authors:** Yin Tianxiu, Zhang Chen, Liu Yuxiang, Zhu Xiaoyue, Hu Jingyao, Guo Haijian, Wang Bei

**Affiliations:** ^1^Department of Epidemiology and Health Statistics, School of Public Health, Southeast University, Nanjing, China; ^2^Zhongda Hospital Affiliated to Southeast University, Nanjing, China; ^3^Department of Integrated Services, Jiangsu Provincial Center for Disease Control and Prevention, Nanjing, China

**Keywords:** dyslipidemia, 25(OH)D, XGBoost, GAM, machine learning

## Abstract

**Introduction:**

Vitamin D is a necessary nutrient that is important for calcium homeostasis and bone health. Dyslipidemia is thought to be a risk factor for the development of atherosclerotic illnesses. Recent research suggests that vitamin D may influence lipid metabolism, specifically the levels of circulating lipids in the blood. However, the relationship between vitamin D and dyslipidemia remains controversial, indicating a need for further research to clarify this association.

**Objectives:**

Data from 780 participants in the “Early Identification, Early Diagnosis Techniques, and Points of Risk for Diabetes in Major Chronic Non-communicable Disease Prevention and Control Studies” were analyzed.

**Methods:**

We employed machine learning with the XGboost algorithm, Least Absolute Shrinkage Selection Operator (LASSO) regression, and univariate logistic regression to screen variables. Subsequently, multiple logistic regression and a generalized additive model (GAM) were utilized to construct models analyzing the association between vitamin D levels and dyslipidemia.

**Results:**

In our study, the XGboost machine learning algorithm explored the relative importance of all included variables, confirming a robust association between vitamin D levels and dyslipidemia. After adjusting for all the important covariates, the results showed that the risk of dyslipidemia in vitamin D insufficiency group and vitamin D deficiency group was 2.11 times and 2.77 times of that in vitamin D sufficiency group, respectively. A smooth curve was constructed based on GAM and a significant negative association was found between 25(OH)D and the risk of dyslipidemia.

**Conclusion:**

There may be a negative association between 25(OH)D and the risk of dyslipidemia. Nonetheless, additional well-designed studies are necessary to substantiate this relationship.

## 1 Introduction

A 25(OH)D level lower than 20 ng/mL is considered vitamin D deficiency. Vitamin D deficiency has become a global public health problem, affecting individuals of all age groups, races and socioeconomic status (1, 2). It is reported that the prevalence of vitamin D deficiency is very high in the United States, Canada and Europe (ranging from 24 to 40%) (3). Beyond its detrimental effects on bone health, inadequate vitamin D levels can lead to a range of chronic diseases, including cardiovascular disease, stroke, and diabetes (4, 5). Dyslipidemia, characterized by elevated levels of triglycerides (TG), total cholesterol (TC), and low-density lipoprotein cholesterol (LDL-C), alongside reduced levels of high-density lipoprotein cholesterol (HDL-C), has been identified as an important risk factor for atherosclerotic diseases such as coronary heart disease, ischemic cerebrovascular disease, and peripheral vascular disease (6, 7). The diagnostic criteria for dyslipidemia are as follows: Satisfy one or more of the following conditions: (1) TC ≥ 6.22 mmol/L; (2) TG ≥ 2.26 mmol/L; (3) HDL-C < 1.04 mmol/L; (4) LDL-C ≥ 4.14 mmol/L (8).

Serum 25(OH)D serves as a sensitive biomarker of vitamin D levels *in vivo* (9). Previous research has linked serum 25(OH)D concentrations with lipid profiles (10, 11). Notably, a study within a Polish cohort found inverse correlations between vitamin D levels and TC, TG, and LDL-C (10). A substantial connection between atherosclerotic lipid profiles and vitamin D deficiency was identified through the analysis of 25 (OH) D levels and other lipid components in 20,000 patients (12). Although various mechanisms have been proposed to elucidate the influence of vitamin D on lipid levels, these effects remain somewhat unclear (13). Considering the significance of blood vitamin D levels, it follows that raising these levels could have a positive impact on the treatment of related conditions such hyperlipidemia in those who are afflicted. Therefore, the relationship between vitamin D levels and dyslipidemia is need to be further explored. The traditional statistical model (logistic regression) simplifies the interaction of variables through linear assumptions and provides clear effect estimates, but fails to capture the potential nonlinear dynamics between vitamin D levels and dyslipidemia. To address these limitations, machine learning has become a powerful tool for modeling complex nonlinear relationships in epidemiology.

Advancements in computer processing power, memory, and storage capacities have enabled machine learning algorithms to efficiently process large data volumes (14). XGBoost, an effective implementation of the gradient boosting decision tree (GBDT) algorithm, has enhanced prediction accuracy, reduced overfitting, and improved algorithm generalization and interpretability, thereby gaining broad acceptance in machine learning and data mining fields (15, 16). We employed a 10-fold cross-validation approach to assess the performance of our data set using the XGBoost model and the LASSO regression algorithm. This involved partitioning the data set into ten folds for cross-validation, with each fold alternately used as a test set while the others served as training sets. The average results from these ten replicates determined the optimal machine learning coefficient. Compared to logistic regression, linear regression better describes the association between two continuous variables while controlling for other confounding factors, but in the case of nonlinear associations, generalized additive model (GAM) is more applicable (17). GAM is a statistical model used to fit data with higher degrees of freedom (18). Before modeling, there is no need to examine the relationship between the response variable and the explanatory variable. Instead, the response variable and each explanatory variable are modeled separately and combined to obtain the GAM. Consequently, this study utilized machine learning with the XGBoost algorithm, LASSO regression, and univariate logistic regression for variable screening. The relationship between vitamin D and dyslipidemia was analyzed using multiple Logistic regression and GAM model.

This study seeks to elucidate the relationship between vitamin D levels and dyslipidemia using a combination of traditional statistical methods and advanced machine learning techniques. By integrating these approaches, we aim to provide a more comprehensive understanding of how vitamin D influences lipid profiles and the potential for its therapeutic modulation in hyperlipidemic conditions. The findings of this study could contribute significantly to the ongoing discussions in nutritional science and clinical practice regarding the management of dyslipidemia and the broader implications of vitamin D deficiency in public health.

## 2 Materials and methods

### 2.1 Study population

The study population was derived from the research project titled “Early identification, early diagnosis techniques and cutting Points of Diabetes risk factors for the Prevention and Control of major Chronic Non-communicable Diseases,” which was conducted among residents aged 18 and elder in Yandu District, Yancheng City, Jiangsu Province from April to July 2018. The investigation encompassed three main components:

1. Epidemiological survey: this included demographic information (e.g., gender, age, and education level), medical history of chronic diseases (e.g., diabetes and hypertension), and behavioral and lifestyle factors (e.g., diet, sleep, exercise, smoking, and alcohol consumption).
2. Physical examination: trained medical personnel measured height, weight, waist circumference, hip circumference, diastolic blood pressure (DBP), systolic blood pressure (SBP), and other indicators.
3. Biochemical indexes testing: venous blood samples were collected after at least 8 h of fasting. Total cholesterol (TC) and high-density lipoprotein cholesterol (HDL-C) tests were performed by Nanjing Adicon Bio-Testing Company. Fasting blood glucose (FPG), low-density lipoprotein cholesterol (LDL-C), triglycerides (TG) and other blood biochemical indicators. Serum 25(OH)D level was measured by electrochemiluminescence.

The inclusion criteria were adults 18 years of age and older. Exclusion criteria were as follows: (1) patients with missing main study indicators (TC, TG, HDL-C, LDL-C); (2) Pregnant women or breastfeeding persons; (3) taking lipid-lowering drugs and vitamin D supplements. Based on the above, a total of 780 subjects were included in the study.

### 2.2 Ethical considerations

All participants understand and consent to the data collection activities. Ethical approval and informed consent signed by participants were obtained prior to data collection.

### 2.3 Evaluation criterion

Vitamin D levels were separated into three groups based on Holick et al.’s (19) reference value. 25(OH)D ≤ 20 ng/mL was considered deficient, 20 ng/mL < 25(OH)D < 29 ng/mL was considered insufficient, and ≥30 ng/mL was considered sufficient.

The criteria for determining dyslipidemia were based on the Chinese Adult Dyslipidemia Prevention and Treatment guidelines for the Chinese population, that is, meeting one or more of the following conditions: (1) TC ≥ 6.2 mmol/L; (2) TG ≥ 2.3 mmol/L; (3) HDL-C < 1.0 mmol/L; (4) LDL-C ≥ 4.1 mmol/L (20).

### 2.4 Statistical analysis

All analyses were performed using SPSS 26 and R 4.3.2. The Mann–Whitney U test, *t*-test, and linear regression were employed for the analysis of continuous variables, while the chi-square test was used for categorical variables. Variable selection was performed using LASSO regression, and their significance was assessed through a multivariate logistic regression model. Generalized additive models (GAM) were utilized to better characterize the association between 25(OH)D levels and dyslipidemia, minimizing the risks of overfitting and underfitting. Finally, the XGBoost algorithm was applied to calculate, rank, and output the most relevant and significant changes in dyslipidemia, ensuring the stability and reliability of the conclusions.

## 3 Results

### 3.1 Characteristics of the study population

There were 780 participants in the study. Statistically significant differences were found in vitamin D levels, BUN, gender, education level, prevalence of hypertension and diabetes, smoking status, and BMI between people with dyslipidemia and people without dyslipidemia (Table 1).

**Table 1:** Characteristics of the included population.

Variables	Dyslipidemia	No dyslipidemia	χ^2^/*t*	*p*
25(OH)D, *n* (%)				
Sufficiency	15 (7.8)	297 (51.2)	10.25	0.01
Insufficiency	83 (43.0)	193 (33.3)		
Deficiency	95 (49.2)	90 (15.5)		
BUN, mmol/L	5.5 ± 1.9	5.2 ± 1.5	−2.20	0.02
CRE, μmol/L	66.5 ± 24.5	65.4 ± 18.5	−0.68	0.50
UA, μmol/L	325.8 ± 79.4	321.7 ± 51.2	−0.61	0.54
Sex, *n* (%)				
Male	107 (55.4)	107 (55.4)	19.63	<0.001
Female	86 (44.6)	86 (44.6)		
Age, *n* (%)				
18–39	18 (9.3)	56 (9.5)	0.10	0.95
40–59	125 (64.8)	373 (63.5)		
≥60	50 (25.9)	158 (26.9)		
Education, *n* (%)				
Junior high school and below	148 (76.7)	488 (83.1)	4.02	0.05
High school and above	45 (23.3)	99 (16.9)		
Smoking, *n* (%)				
Yes	61 (31.6)	130 (22.1)	7.03	0.01
No	132 (68.4)	457 (77.9)		
Alcohol, *n* (%)				
Yes	57 (29.5)	137 (23.3)	2.98	0.08
No	136 (70.5)	450 (76.7)		
Hypertension, *n* (%)				
Yes	85 (44.0)	240 (40.9)	13.35	<0.001
No	108 (56.0)	347 (59.1)		
Diabetes, *n* (%)				
Yes	40 (20.7)	67 (11.4)	10.64	<0.05
No	153 (79.3)	520 (88.6)		
BMI, kg/m^2^				
−24.9	67 (34.7)	328 (55.9)	31.03	<0.001
25–29.9	98 (50.8)	223 (38.0)		
30-	28 (14.5)	36 (6.1)		

### 3.2 Single factor analysis: univariate logistic regression

Univariate logistic regression was used to analyze the correlation between dyslipidemia and serum 25(OH)D level, sex, age, education level, smoking, alcohol consumption, BMI, hypertension, and diabetes. There was a positive correlation between BMI levels and dyslipidemia. Men have a greater risk of dyslipidemia than women. High school and above, no hypertension, and no diabetes were negatively correlated with dyslipidemia (Table 2).

**Table 2:** Univariable analysis for dyslipidemia.

Variables	OR (95%CI)	*p*-value
Sex		
Female	Reference	
Male	1.85 (1.14, 2.99)	0.01
Age		
18–39	Reference	
40–59	1.71 (0.82, 3.57)	0.16
≥60	1.17 (0.77, 1.77)	0.46
Education		
Junior high school and below	Reference	
High school and above	0.63 (0.40, 1.00)	0.05
Smoking		
No	Reference	
Yes	1.14 (0.71, 1.85)	0.59
Alcohol		
Yes	Reference	
No	0.69 (0.43, 1.11)	0.13
BMI (kg/m^2^)	1.12 (1.06, 1.17)	<0.001
25(OH)D (ng/mL)		
Sufficiency	Reference	
Insufficiency	2.11 (1.08, 4.14)	0.03
Deficiency	2.87 (1.46, 5.62)	0.00
Hypertension		
Yes	Reference	
No	0.60 (0.41, 0.88)	0.01
Diabetes		
No	Reference	
Yes	0.54 (0.33, 0.88)	0.01
BUN (mmol/L)	1.08 (0.95, 1.21)	0.23
CRE (μmol/L)	1.00 (0.99, 1.01)	0.90
UA (μmol/L)	1.00 (0.99, 1.00)	0.60

BMI, body mass index; BUN, Blood Urea Nitrogen; CRE, Creatinine; UA, Uric acid.

### 3.3 Multivariate analysis: LASSO regression

**Figure 1:**
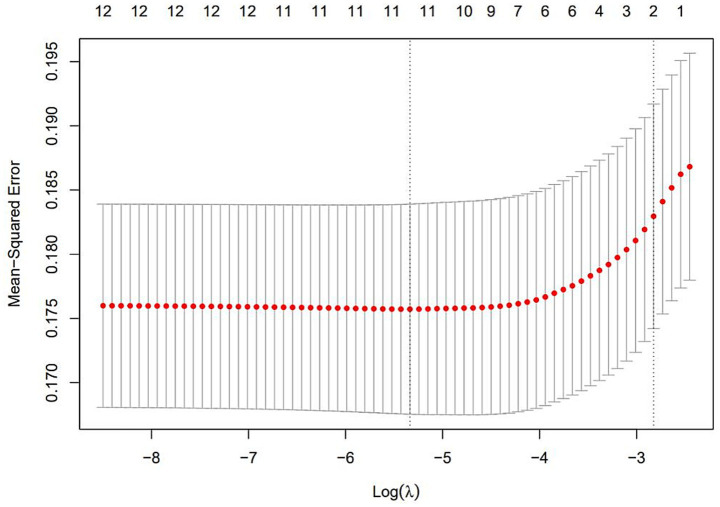
Lasso coefficient of variables in the model.

**Figure 2:**
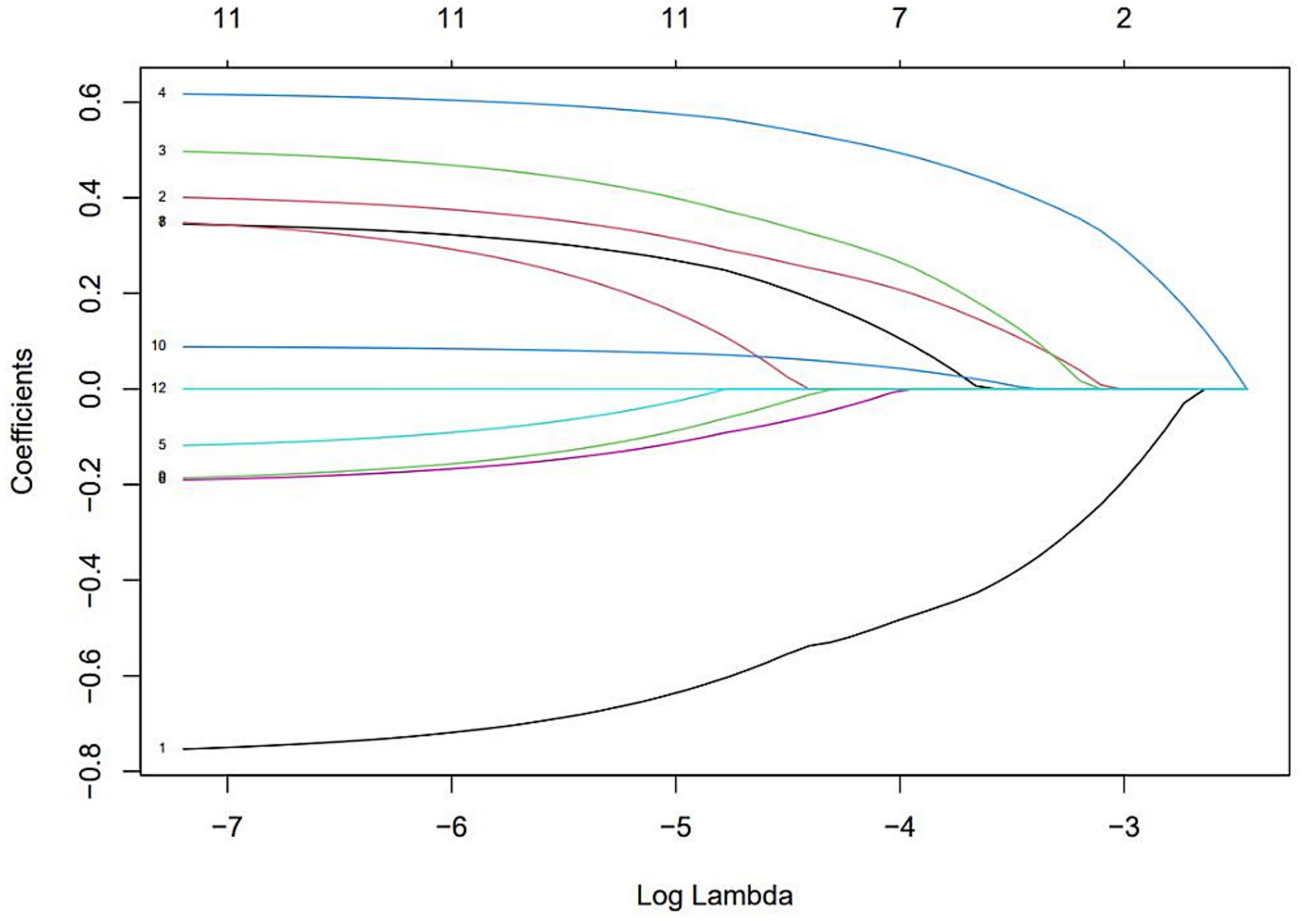
The optimal penalty coefficient [Lambda (log) = 0.005281067 (−5.243627)] in the Lasso regression was identified with the minimum criterion.

**Figure 3:**
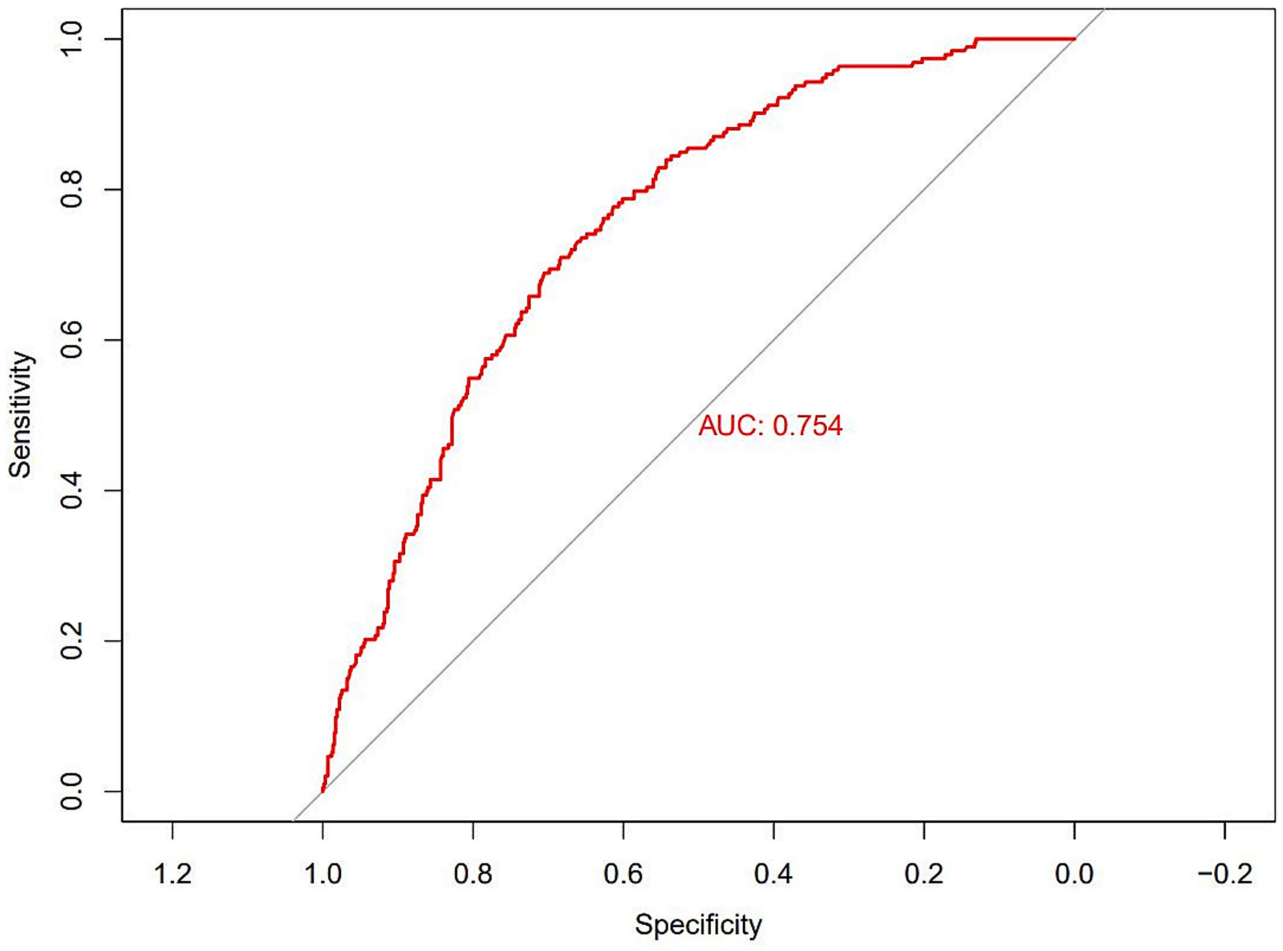
Receiver operating characteristic (ROC) curves according to LASSO regression.

The results of selecting variables using LASSO regression are as follows. In Figure 1, the red dots represent the adjustment parameter (Lambda), and the two dashed lines represent the two special Lambda(log) values selected in the LASSO model using 10x cross-validation. That is, Lambda(log) values [lambda. Min(log)] and larger lambda(log) values are given for the minimum average cross-validation error so that the error is within 1 standard error of the minimum [lambda.1se (log)]. The two lambda values select variables that are included in the corresponding optimization model. The Lambda minimum (log) is 0.005281067 (−5.243627) (Figure 2). The AUC value of the receiver operating characteristic (ROC) curve of the prediction model based on variables screened by Lambda values was 0.754 (Figure 3), indicating that LASSO regression can effectively screen out variables strongly associated with dyslipidemias.

According to the 10-fold cross-verified Lambda min (log), the selected variables are sex, hypertension, diabetes, BMI, age, 25(OH)D, education, alcohol, smoking, BUN, and UA (Supplementary Table 1). The variables screened by LASSO regression were included in multivariate logistic regression. Compared with the vitamin D sufficiency group, the risk of dyslipidemia was 2.11 times higher in the vitamin D insufficiency group and 2.77 times higher in the vitamin D deficiency group (Table 3).

**Table 3:** The association between 25(OH)D and Dyslipidemia in the multiple regression model.

25(OH)D status	OR (95%CI)	*p*-value
Sufficiency	Reference	
Insufficiency	2.11 (1.15, 3.85)	0.02
Deficiency	2.77 (1.50, 5.11)	<0.001

### 3.4 Generalized additive model

**Figure 4:**
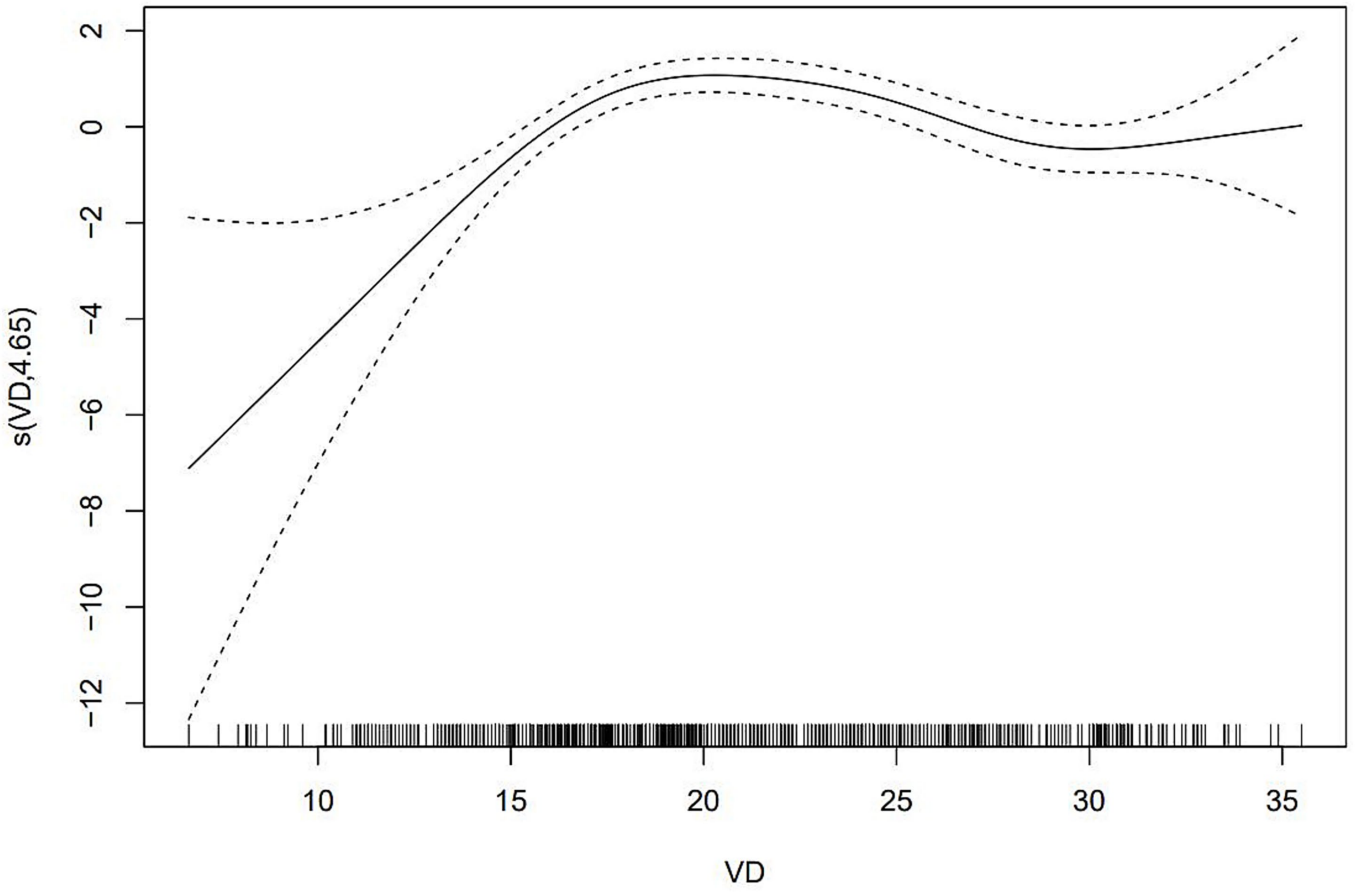
The relationship between 25(OH)D and dyslipidemia according to GAM.

After adjusting for the statistically significant variables of LASSO regression screening in GAM, 25(OH)D levels were found to be significantly negatively associated with the risk of dyslipidemia (Figure 4). The risk of dyslipidemia decreased rapidly as vitamin D levels increased, and the risk of dyslipidemia stabilized when vitamin D levels were above 30 ng/mL.

### 3.5 Machine learning using the XGBoost algorithm model

**Figure 5:**
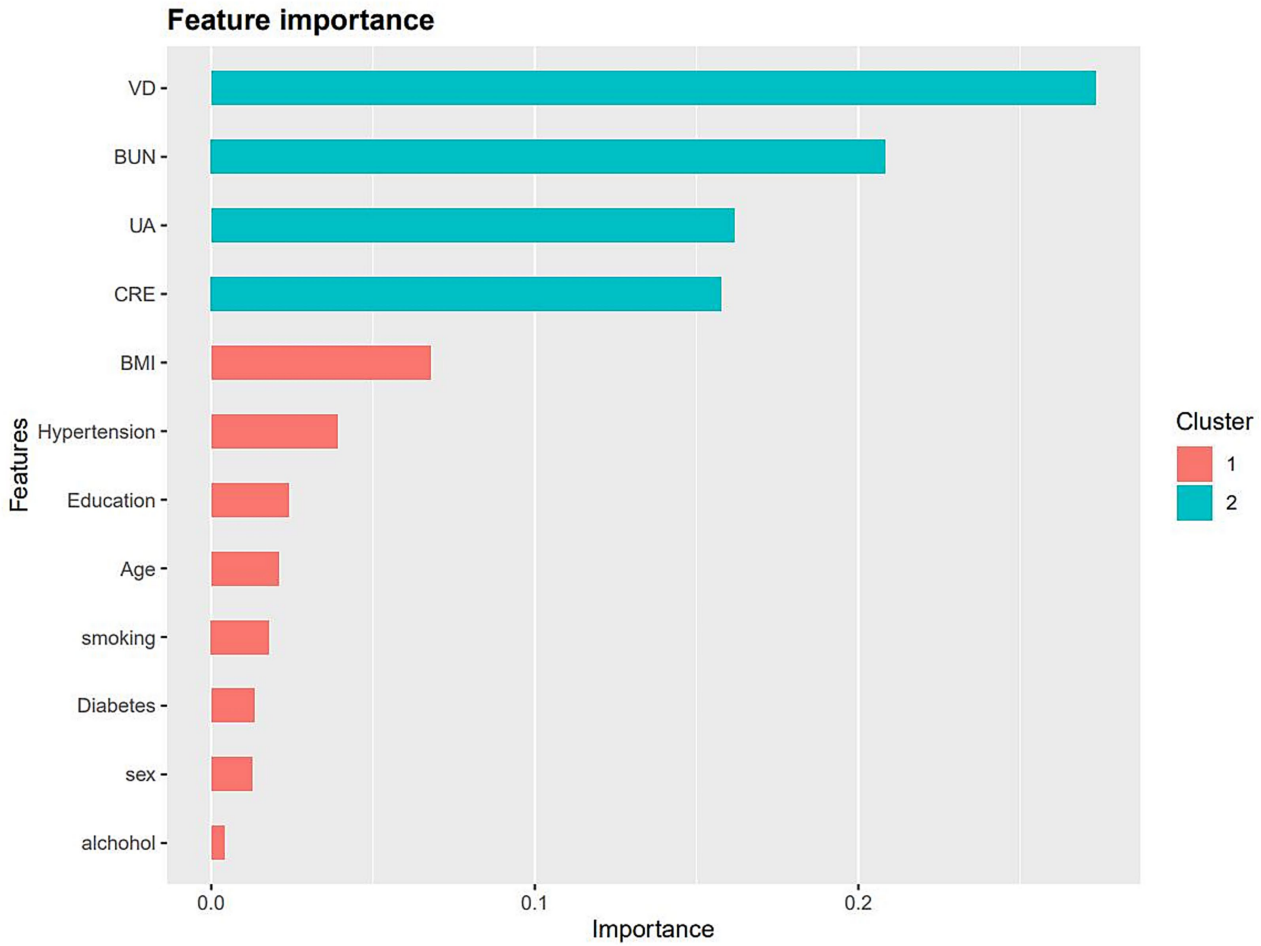
The relative importance of the selected variables using XGBoost and the corresponding variable importance score. The *x*-axis indicates the importance score, which is the relative number of a variable that is used to distribute the data, *y*-axis indicates the selected variable.

We input all the variables into the XGboost machine learning algorithm, which is based on a 10-fold cross-validation approach for dyslipidemia. These variables included sociodemographic data of the participants and all relevant laboratory data. Based on the relative importance of the XGboost algorithm’s additional variables, 25(OH)D, BUN, UA, CRE, BMI, and hypertension are the six most important variables in the dataset (Figure 5).

## 4 Discussion

Dyslipidemia and vitamin D deficiency are two significant global health challenges, each contributing to a wide array of diseases and increased mortality. Dyslipidemia, particularly elevated plasma LDL-C levels, is a well-established risk factor for CVD (21). Recent studies indicates that dyslipidemia is significantly associated with an increased risk of mortality from various conditions, including diabetes, malignancies, systemic autoimmune diseases, and kidney disease (22–25). Epidemiological evidence further confirms that dyslipidemia is both a recognized and modifiable risk factor for CVD, emphasizing the importance of effective lipid management to prevent cardiovascular events and reduce the global burden of cardiovascular disease (26).

Similarly, vitamin D deficiency represents a pervasive health issue that affects populations worldwide and is linked to numerous diseases, including cardiovascular disease (27, 28). Extensive evidence supports the association between lower vitamin D levels and an increased risk of conditions such as osteoporosis, chronic kidney disease, autoimmune diseases, neurological disorders, and specific types of cancer (29–32). Furthermore, consistent findings reveal a strong relationship between reduced vitamin D levels and increased risk of death. Like dyslipidemia, serum vitamin D levels significantly influence the development and progression of CVD and diabetes. Adequate levels of vitamin D have been shown to protect the cardiovascular system and help prevent diseases such as hypertension, coronary artery disease, and stroke (4). In conclusion, addressing these two critical health challenges can significantly improve population health and reduce the burden of chronic diseases.

In this study, vitamin D was identified as a significant protective factor against dyslipidemia based on the relative importance of variables analyzed using the XGBoost algorithm. Univariate and multivariate logistic regression analyses confirmed that vitamin D insufficiency and deficiency significantly increased the risk of dyslipidemia. The generalized additive model (GAM) further demonstrated a nonlinear association between vitamin D levels and dyslipidemia risk, with a rapid decline in risk as vitamin D levels increased. This trend stabilized when serum vitamin D levels exceeded 30 ng/mL. These findings align with previous studies, such as that of Ying Wang et al., which reported an increased risk of dyslipidemia associated with vitamin D deficiency (11). Similarly, a cross-sectional study of 3,788 Chinese adults found that 25(OH)D levels were negatively correlated with triglycerides (*β* = −0.077, *p* < 0.05) and LDL cholesterol (*β* = −0.245, *p* < 0.05) (33). Globally, more than 50% of the population has vitamin D levels below 30 ng/mL, affecting over 1 billion children and adults (1). Given the potential adverse effects of vitamin D deficiency on lipid metabolism and cardiovascular health, our findings highlight the importance of prioritizing vitamin D screening in high-risk populations with serum levels below 30 ng/mL. BMI showed a positive correlation with dyslipidemia risk, consistent with findings from other prospective cohort studies. These studies demonstrated that increased BMI and obesity significantly contribute to dyslipidemia risk (34–36). Additionally, men displayed a higher risk of dyslipidemia, potentially due to smoking-related factors. Nicotine and other harmful substances in tobacco smoke raise free fatty acid levels in the blood, promoting hepatic triglyceride synthesis and reducing lipoprotein lipase activity. This leads to elevated triglyceride levels and reduced HDL-C production (37–39).

The mechanisms underlying the effect of vitamin D on lipid levels remain unclear, although several hypotheses have been proposed. First, vitamin D may inhibit hepatic triglyceride synthesis and secretion by enhancing intestinal calcium absorption (40). Vitamin D may also regulate calcium metabolism and increase intestinal calcium absorption, thereby reducing fatty acid absorption (11). Second, elevated serum calcium levels can enhance fecal fat excretion and bile acid secretion, leading to reduced cholesterol levels (41, 42). Third, high levels of parathyroid hormone (PTH) may lead to elevated TG, while 25(OH)D can inhibit serum PTH secretion (43, 44). Specifically, adequate vitamin D will increase the calcium level within fat cells, enhance the activity of fatty acid synthase, inhibit fat breakdown, and increase the storage of lipids within fat cells (45). Moreover, vitamin D can inhibit the migration and phagocytic activity of macrophages, thereby reducing the deposition of low-density lipoprotein cholesterol (LDL-C) (46). Therefore, vitamin D can affect TG concentration by regulating parathyroid hormone levels. Additionally, vitamin D deficiency has been linked to insulin resistance and impaired beta-cell activity, both of which disrupt lipoprotein metabolism, resulting in elevated triglycerides and reduced HDL-C levels (47, 48). Finally, vitamin D may regulate genes involved in cholesterol synthesis and clearance, such as CYP7A1 and ABCA1, and also regulate the inflammatory pathways that play a crucial role in lipid metabolism (49).

Compared with previous studies, this research has several advantages. First, it is based on a real-world population study, providing practical relevance. Second, it utilizes robust statistical techniques such as the XGBoost algorithm and LASSO regression for variable screening and validation. The XGBoost model is an algorithm based on decision tree integration and gradient boosting, which solves the problem of class imbalance through sample weighting and class weighting mechanisms and has powerful functions for handling high-dimensional features and capturing nonlinear relationships. Additionally, the use of the GAM model to analyze serum vitamin D levels as a continuous variable provided insights into the nonlinear relationship between vitamin D levels and dyslipidemia, avoiding overfitting and underfitting. GAM extends the traditional regression model, allowing predictor variables to flexibly influence the results, using a data-driven curve fitting approach instead of assuming a fixed logarithmic linear form. Despite these strengths, the study has limitations. First, in the data collection process, factors that may affect vitamin D levels, such as daily diet, sun exposure duration, and sunscreen use, were not taken into account. These factors may interfere with the research results and affect the accuracy of the conclusions. In addition, since the study population is based on Chinese data, the general applicability of these results in other countries and regions requires further research. Due to its cross-sectional study nature, it is impossible to determine the causal relationship. Future prospective cohort studies are needed to confirm these findings. Nonetheless, this study provides valuable insights into the relationship between vitamin D status and dyslipidemia. Further research, including cohort and experimental studies, is necessary to explore the causal mechanisms linking vitamin D levels to dyslipidemia.

## 5 Conclusion

Dyslipidemia is an important risk factor for cardiovascular diseases, so effective control of lipid levels is crucial for preventing cardiovascular events and reducing the associated medical burden. In this study, based on the XGBoost algorithm, vitamin D was identified as an important protective factor for preventing dyslipidemia. Univariate and multivariate logistic regression analysis confirmed that insufficient and deficient vitamin D levels significantly increase the risk of dyslipidemia. The GAM model further indicated that there is a non-linear association between vitamin D levels and the risk of dyslipidemia, that is, individuals with insufficient or deficient vitamin D levels seem to have a higher risk of dyslipidemia than those with adequate vitamin D levels. When the serum vitamin D level is below 30 ng/mL, targeted screening for high-risk populations may help prevent dyslipidemia and its adverse cardiovascular consequences. These findings emphasize the importance of incorporating vitamin D status into the lipid dyslipidemia prevention strategy, and also indicate the need for more research, including cohort studies and experimental studies, to verify and deepen our understanding of this association.

## Data Availability

The datasets presented in this article are not readily available because due to the nature of this research, participants of this study did not agree for their data to be shared publicly, so supporting data is not available. Requests to access the datasets should be directed to 1060581879@qq.com.
