# Supplementary material for: Exploring the association between vitamin D levels and dyslipidemia risk: insights from machine learning and generalized additive models
Source: Front Nutr. 2025 Aug 11;12:1618610. doi: 10.3389/fnut.2025.1618610 (PMC12375490; doi:10.3389/fnut.2025.1618610)
Supplement: Supplementary file 1 [file Table_1.docx]

Supplementary Material

# Supplementary Tables

# Supplementary Table 1. The selected variables

# according to the 10-fold cross-verified Lambda min (log)

| **LASSO Regression** | |
| --- | --- |
| Coefficients |  |
| Lambda (log) | 0.005281067 (-5.243627) |
| (Intercept) | 0.1103514 |
| Sex | -0.1174732 |
| Hypertension | 0.05923946 |
| Diabetes | 0.08576444 |
| BMI(kg/m2) | 0.1077124 |
| Age | -0.01191065 |
| 25(OH)D (ng/mL) | -0.02278232 |
| Education | 0.0518913 |
| alchohol | 0.03570302 |
| smoking | -0.01930433 |
| BUN (mmol/L) | 0.01443789 |
| CRE (μmol/L) | 0 |
| UA (μmol/L) | 3.61219E-05 |

Abbreviations: body mass index, BMI; Blood Urea Nitrogen, BUN; Creatinine, CRE; Uric acid, UA.
